# A prospective birth cohort study of different risk factors for development of allergic diseases in offspring of non-atopic parents

**DOI:** 10.18632/oncotarget.14565

**Published:** 2017-01-09

**Authors:** Ming-Tsung Lee, Chih-Chiang Wu, Chia-Yu Ou, Jen-Chieh Chang, Chieh-An Liu, Chih-Lu Wang, Hau Chuang, Ho-Chang Kuo, Te-Yao Hsu, Chie-Pein Chen, Kuender D. Yang

**Affiliations:** ^1^ Research Assistant Center, Show Chwan Memorial Hospital, Changhua, Taiwan; ^2^ Institute of Clinical Medicine, National Yang-Ming University, Taiwan; ^3^ Department of Pediatrics, Po-Jen Hospital, Kaohsiung, Taiwan; ^4^ Departments of Pediatrics and Medical Research, Kaohsiung Chang Gung Memorial Hospital, Kaohsiung, Taiwan; ^5^ Chang Gung University College of Medicine, Kaohsiung, Taiwan; ^6^ Department of Obstetrics and Gynecology, Kaohsiung Chang Gung Memorial Hospital, Taiwan; ^7^ Chang Gung University College of Medicine, Kaohsiung, Taiwan; ^8^ Department of Medical Research, MacKay Memorial Hospital, Taipei; ^9^ Department of Pediatrics, MacKay Memorial Hospital, Taipei; ^10^ Institute of Biomedical Sciences, MacKay Medical College, New Taipei, Taiwan

**Keywords:** birth cohort, atopic dermatitis, allergic rhinitis, asthma, perinatal environment

## Abstract

Background: Allergic diseases are thought to be inherited. Prevalence of allergic diseases has, however, increased dramatically in last decades, suggesting environmental causes for the development of allergic diseases.

Objective: We studied risk factors associated with the development of atopic dermatitis (AD), allergic rhinitis (AR) and asthma (AS) in children of non-atopic parents in a subtropical country.

Methods: In a birth cohort of 1,497 newborns, parents were prenatally enrolled and validated for allergic diseases by questionnaire, physician-verified and total or specific Immunoglobulin E (IgE) levels; 1,236 and 756 children, respectively, completed their 3-year and 6-year follow-up. Clinical examination, questionnaire, and blood samples for total and specific IgE of the children were collected at each follow-up visit.

Results: Prevalence of AD, AR and AS was, respectively, 8.2%, 30.8% and 12.4% in children of non-atopic parents. Prevalence of AR (*p*<.001) and AS (*p*=.018) was significantly higher in children of parents who were both atopic. A combination of Cesarean section (C/S) and breastfeeding for more than 1 month showed the highest risk for AD (OR=3.111, *p*=.006). Infants living in homes with curtains and no air filters had the highest risk for AR (OR=2.647, *p*<.001), and male infants of non-atopic parents living in homes without air filters had the highest risk for AS (OR=1.930, *p*=.039).

Conclusions: Breastfeeding and C/S affect development of AD. Gender, use of curtains and/or air filters affect AR and AS, suggesting that control of the perinatal environment is necessary for the prevention of atopic diseases in children of non-atopic parents.

## INTRODUCTION

Prevalence of allergic diseases, including atopic dermatitis (AD), allergic rhinitis (AR), and asthma (AS) has increased dramatically in the last decades and has raised concern over the influence of lifestyle and environmental factors on the development of allergic diseases [1]. Allergic disease has been thought as an inherited disease. Parents with allergies raise offspring with a higher incidence rate of allergic diseases. Many studies have investigated the influence of parental atopy on the allergic disorders of their offspring. For example, maternal history [2], paternal history [3], maternal IgE levels [4], paternal IgE levels [5], cord blood IgE levels [6], and genes [7] were associated with the development of allergic sensitization and diseases. Many studies have also explored the impacts of extrinsic environmental factors on children's allergic disorders. For instance, indoor [8] and outdoor [9] allergens, indoor [10] and outdoor [11] air pollutants, passive smoking [12], food [13], breastfeeding [14], pets [15], furniture fabrics [16], were potentially connected with the development of the offspring's allergic sensitization and diseases. However, the relationships are still unclear.

In a birth cohort study, we found that the interactions between genes and environmental factors on blood IgE levels began during the prenatal stage [17], and the genes associated with higher blood IgE levels were different among infants, toddlers, and children [7]. We also examined whether partially hydrolyzed infant formula decreased allergic sensitization or allergic diseases, and found that partially hydrolyzed infant formula reduced the risks of food sensitization before three years of age, but the prevalence of allergic diseases could not be reduced [18]. A study of allergic diseases in school age children reported that the history of lower respiratory infection in infancy was associated with a higher incidence of asthma in children of non-atopic parents. These results support that children from non-atopic parents could develop atopic diseases under different environmental influences [19]. A Finnish cohort study also demonstrated that children from atopic parents without mold exposure had higher incidence rate ratios (IRRs) of 1.54 (95% CI, 1.09-2.18) than those from non-atopic parents without exposure to mold. On the other hand, the risk of mold exposure in children with non-atopic parents was also increased with an IRR of 2.56 (95% CI, 0.93-7.08), corresponding to reference group from non-atopic parents without mold exposure [20]. Given that, it is implied that heredity is a strong determinant of childhood asthma, and environmental exposure also increased the risk of childhood asthma.

In the birth cohort follow-up, we attempted to examine the environmental risk factors for the development of allergic disorders in children of non-atopic parents in Taiwan. We compared the prevalence of allergic disorders and analyzed the distribution of the risk factors among 3 groups of children according to different parental atopy (both, one, or neither atopic). We then focused on analyses of potential risk factors for the development of AD, AR, and AS in children of non-atopic parents.

## RESULTS

### Demographic data of children with and without completion of follow-up

In total, 1,848 newborns were recruited, 1,629 infants were followed at least once. Of these newborns born in the hospital, 1497 received CBIgE measurement, 1,236 and 756 children completed 3-year-old and 6-year-old follow-ups, and 737 children had all charts and blood tests for analysis. Of the 737 children, 380 children were neither parents atopic, 293 children were one parent atopic, and 64 were both parents atopic. The demographic data and baseline parental characteristics between children with and without completion of 6-year-old follow-up revealed no significant differences (Table 1).

**Table 1:** Basic characteristics of children with and without completion of 6-year-old follow-up

		Complete		Incomplete				
		*N*	%	*N*		%	X^2^	*p*
Gender							0.000	1.000
	Female	354	46.8%	463		46.9%		
	Male	402	53.2%	524		53.1%		
	Total	756	100.0%	987		100.0%		
Delivery							2.391	.122
	NSD	554	73.7%	587		70.0%		
	C/S	198	26.3%	251		30.0%		
	Total	752	100.0%	838		100.0%		
Preterm							.469	.493
	>=37weeks	704	93.1%	947		94.0%		
	<37 weeks	52	6.9%	60		6.0%		
	Total	756	100.0%	1007		100.0%		
Mother AD							.475	.491
	No	635	84.0%	872		82.7%		
	Yes	121	16.0%	183		17.3%		
	Total	756	100.0%	1055		100.0%		
Mother AR							.018	.893
	No	431	57.0%	606		57.4%		
	Yes	325	43.0%	449		42.6%		
	Total	756	100.0%	1055		100.0%		
Mother AS							.227	.634
	No	695	91.9%	962		91.2%		
	Yes	61	8.1%	93		8.8%		
	Total	756	100.0%	1055		100.0%		
Mother History							.070	.792
(AD, AR	No	351	46.4%	482		45.7%		
and/or AS)	Yes	405	53.6%	573		54.3%		
	Total	756	100.0%	1055		100.0%		
Father AD							0.000	1.000
	No	677	89.6%	939		89.6%		
	Yes	79	10.4%	109		10.4%		
	Total	756	100.0%	1048		100.0%		
Father AR							.999	.318
	No	498	65.9%	715		68.2%		
	Yes	258	34.1%	333		31.8%		
	Total	756	100.0%	1048		100.0%		
Father AS							0.000	1.000
	No	716	94.7%	993		94.8%		
	Yes	40	5.3%	55		5.2%		
	Total	756	100.0%	1048		100.0%		
Father History							1.254	.263
(AD, AR	No	417	55.2%	607		57.9%		
and/or AS)	Yes	339	44.8%	441		42.1%		
	Total	756	100.0%	1048		100.0%		
Mother allergen							.471	.492
sensitization	Negative	501	67.1%	615		65.4%		
	Positive	246	32.9%	326		34.6%		
	Total	747	100.0%	941		100.0%		
Father allergen							3.232	.072
sensitization	Negative	403	54.4%	550		58.9%		
	Positive	338	45.6%	384		41.1%		
	Total	741	100.0%	934		100.0%		
				Incomplete				
	***N***	Mean	SD	***N***	Mean	SD	t	***p***
Gestational age	756	38.62	1.544	1007	38.66	1.520	-.521	.603
Cord Blood IgE	657	0.32	0.791	840	0.40	1.563	−1.074	.283
Log Maternal IgE	750	1.63	.577	936	1.63	.588	.002	.998
Log Paternal IgE	743	1.82	.560	935	1.81	.563	.469	.639

### The prevalence of allergic diseases in children with and without atopic parents

Among the 380 children of non-atopic parents, 8.2% of the children had AD, 30.8% of the children had AR, and 12.4% of the children had AS. Among the 293 children of one parent atopic, 11.6 % of the subjects had AD, 39.9 % of the children had AR, and 17.4 % of the children had AS. The third group consisting of 64 children of atopic parents revealed a prevalence of AD at 9.4%, AR at 54.7%, and AS at 25.0%. The prevalence of AD among the 3 groups of subjects was not significantly different (X^2^=2.262, p=.323). The prevalence of AR was significantly higher in children of parents who were both atopic (X^2^=15.969, p<.001). The prevalence of AS was also significantly higher in children whose parents were atopic (X^2^=8.081, p=.018) (Table 2). Airway allergic diseases (AR and AS), but not AD, were significantly higher in children from atopic parents suggesting they are two disease entities in term of different inheritance. Moreover, patients with AS have more than 80% (86.3%) of comorbidity with AR, but have less than 25% (23.1%) comorbidity with AD.

**Table 2:** Prevalence of atopic dermatitis (AD), allergic rhinitis (AR), and asthma (AS) in children from both atopic, either one atopic and both non-atopic parents

	Children from Both Non-Atopic Parents	Children from One Atopic Parent	Children from Atopic Parents	X^2^	*P*
AD	8.2%	11.6%	9.4%	2.262	.323
AR	30.8%	39.9%	54.7%	15.969	< .001
AS	12.4%	17.4%	25.0%	8.081	.018

Abbreviations: AD: Atopic Dermatitis; AR: Allergic Rhinitis; AS: Asthma.

### The demographic factors in children with and without atopic parents

We analyzed the distribution of perinatal environmental factors among children of non-atopic parents, one atopic parent, and atopic parents. A majority of the perinatal environmental factors had similar distributions among the three groups of children (Table 3). The proportion of gender, preterm delivery, family member smoking, cesarean section (C/S) or first parity was not significantly different among the 3 groups. The study site was located in a subtropical region so that more than two-thirds of families used curtains for window coverings and tile floor, and the proportion among the 3 groups was not significantly different. Similarly, some families used dehumidifiers and/or air filters in the subtropical country. Families where both parents were atopic appeared to have a higher rate of air filter use (22.5, 19.4, and 18.3%; both atopic, one atopic and non-atopic) and dehumidifier (18.3, 15.9 and 15.7%; both atopic, one atopic and non-atopic) use, although there were no significant differences among the three groups. In contrast, a pet (12.5-14.4%) or carpeting (6.5%-7.5%) in the home was not common in the families of this cohort. About one third of the participants in this cohort had wooden floors.

**Table 3:** The distribution of the risk factors in children from non-atopic and atopic parents

	Both Non-Atopic	Either Atopic	Both Atopic	X^2^	*P*
Gender (Male)	55.5%	50.8%	50.8%	3.607	.165
Preterm	6.0%	7.0%	5.9%	.650	.723
Smoking	23.3%	21.5%	22.0%	.622	.733
C/S	27.9%	27.9%	26.4%	.111	.946
First Parity	52.3%	56.2%	52.4%	2.159	.340
Housing (Townhouse)	63.1%	65.6%	56.2%	3.505	.173
Dehumidifier	15.7%	15.9%	18.3%	.563	.755
Air Filter	18.3%	19.4%	22.5%	1.318	.517
Curtaining	82.9%	80.7%	80.8%	1.280	.527
Tile Floor	70.1%	70.3%	60.8%	4.615	.100
Wooden Floor	36.8%	32.6%	33.3%	2.995	.224
Carpeting	6.5%	6.6%	7.5%	.165	.921
Pet at Home	14.4%	14.2%	12.5%	.300	.861
Breast Feeding ≥1 month	63.6%	70.1%	72.6%	8.384	.015

More than three-fifths of the mothers breastfed their infants in the first month, and then stop or supplemented with infant formula. We analyzed the influence of breastfeeding by using a cut-off at equal to or more than one month of breastfeeding. Among the three study groups, children from the non-atopic parents had a significantly lower rate of breastfeeding over one month with 63.6%, in comparison with children from atopic parents (72.6%) or one atopic parent (70.1%) (X^2^=8.384, p=.015). This bias might be related to recognition of the value of breastfeeding in the prevention of allergic diseases by atopic parents. Umbilical cord blood IgE (CBIgE) level cut-off 0.35 kU/L was significantly higher in newborns from atopic parents than those from non-atopic parents (X^2^=14.885, p=.001); however, the elevated CBIgE level could not predict childhood AD (p=.184), AS (p=.425) or AR (p=.090).

### Risk factors for development of AD, AR or AS in children from non-atopic parents

In AD, we initially analyzed the relationships between perinatal risk factors and development of AD at 6 years of age for all subjects (including both parents with atopy, one parent with atopy, and both parents without atopy) in the cohort. In univariate analysis (UVA) for all subjects in the cohort, we found that breastfeeding for more than 1 month (OR=1.81, p=.050) was borderline significant associated with AD. In the multivariate logistic regression analysis (MVA) for all subjects in the cohort, no risk factor was significantly associated with AD. Breastfeeding for more than 1 month approached borderline significance (OR=2.06, p=.051) in predicting AD and air filter use reached borderline significance in predicting protection from AD (OR=0.43, p=.053). In contrast, we found that breastfeeding for more than 1 month (OR=3.02, p=.027) and dehumidifier use (OR=2.40, p=.039) were significantly associated with development of AD in children of parents without atopy in UVA. While controlling other risk factors in MVA, breastfeeding for more than 1 month remained a significant predictor for AD (OR=3.43, p=.040), and C/S was also a significant risk factor for AD (OR=2.66, p=.042) in children from non-atopic parents.

In AR, we found that male gender was a significant risk factor for AR (OR=1.59, p=.009), while preterm birth and living in a townhouse were significant protective factors for AR (OR=0.51, p=.044 and OR=.071, p=.037, respectively) in UVA. In MVA for AR, male gender remained a significant predictor (OR=1.59, p=.009) and preterm birth and living in a townhouse were significantly protective factors for AR (OR=0.46, p=.046 and OR=0.68, p=.034, respectively). In children of parents without atopy, male gender (OR=1.77, p=.013) and use of curtains (OR=2.06, p=.035) were significant risk factors for AR; and preterm birth (OR=0.29, p=.047) and air filter use (OR=0.48, p=.008) were significantly protective factors in UVA. While controlling other risk factors in MVA, male gender (OR=2.08, p=.011), family member smoking (OR=2.02, p=.021), use of curtains (OR=2.72, p=.025) and tile floor (OR=2.28, p=.013) were significant risk factors for AR of children from both non-atopic parents.

In prediction of AS, UVA analyses found that male gender was a borderline significant predictor for AS (OR=1.50, p=.050) in all children studied. In MVA, none of the risk factors reached a statistically significant relationship with AS. Results of children from both non-atopic parents also showed no risk factor significantly associated with development of AS (Table 4). This suggests that there is no single factor strong enough to affect the development of AS in children with and without atopic parents. We next analyzed whether 2 or 3 combined coexisting factors were associated with the development of allergic diseases.

**Table 4-1:** The relationships between independent variables and atopic dermatitis

	Both Non-Atopic Parents				All Children			
	UVA		MVA		UVA		MVA	
	OR	*P*	OR	*P*	OR	*P*	OR	*P*
Gender (Male)	.813	.582	.937	.883	.924	.750	.919	.769
Preterm	.000	.998	.000	.998	.563	.345	.602	.422
Smoking	1.142	.749	1.135	.796	1.136	.646	1.462	.224
C/S	1.838	.117	2.657	.042	1.596	.076	1.565	.153
First Parity	.783	.536	.662	.355	1.146	.607	. 907	. 737
Housing (Townhouse)	.777	.529	.821	.667	.898	.689	. 899	. 726
Dehumidifier	2.404	.039	2.332	.128	1.593	.135	1.305	.480
Air Filter	.915	.852	.530	.275	.693	.284	.432	.053
Use of curtains	1.036	.944	1.039	.952	1.053	.873	1.035	.928
Tile Floor	1.106	.807	.994	.990	1.141	.634	1.434	.285
Wooden Floor	1.817	.114	1.786	.204	1.417	.166	1.379	.276
Carpeting	.435	.422	.000	.998	1.061	.904	1.325	.621
Pet	1.563	.326	1.560	.439	1.313	.390	1.284	.501
Breast Feeding ≥1m	3.023	.027	3.425	.040	1.809	.050	2.055	.051

Abbreviations: UVA: Univariate Analysis; MVA: Multivariate Analysis.

**Table 4-2:** The relationships between independent variables and allergic rhinitis

	Both Non-Atopic Parents				All Children			
	UVA		MVA		UVA		MVA	
	OR	*P*	OR	*P*	OR	*P*	OR	*P*
Gender (Male)	1.770	.013	2.078	.011	1.565	.004	1.591	.009
Preterm	.288	.047	.346	.126	.506	.044	.462	.046
Smoking	1.528	.084	2.022	.021	1.172	.357	1.291	.201
C/S	.989	.964	1.151	.659	.925	.652	1.038	.856
First Parity	.797	.336	. 651	. 131	1.030	.854	. 826	. 287
Housing (Townhouse)	.681	.114	. 807	. 471	.709	.037	. 675	. 034
Dehumidifier	.524	.062	.485	.098	.761	.217	.687	.144
Air Filter	.427	.008	.496	.064	.682	.052	.725	.161
Use of curtains	2.062	.035	2.722	.025	1.146	.491	1.191	.450
Tile Floor	1.500	.108	2.275	.013	1.150	.402	1.311	.167
Wooden Floor	1.330	.210	1.476	.171	.986	.927	.958	.817
Carpeting	.530	.215	.596	.463	.734	.334	.952	.892
Pet at Home	1.023	.939	1.148	.726	1.011	.958	1.106	.675
Breast Feeding ≥1m	.879	.581	.868	.633	.994	.968	1.113	.585

Abbreviations: UVA: Univariate Analysis; MVA: Multivariate Analysis.

**Table 4-3:** The relationships between independent variables and asthma

	Both Non-Atopic Parents				All Children			
	UVA		MVA		UVA		MVA	
	OR	*P*	OR	*P*	OR	*P*	OR	*P*
Gender (Male)	1.770	.085	1.514	.266	1.498	.050	1.360	.178
Preterm	.599	.497	.805	.792	.562	.232	.750	.568
Smoking	1.199	.597	1.170	.696	1.365	.159	1.348	.226
C/S	.716	.375	.621	.296	.790	.326	.686	.176
First Parity	1.011	.973	.782	.508	1.093	.672	.962	. 865
Housing (Townhouse)	1.530	.249	1.753	.181	1.228	.357	1.167	. 526
Dehumidifier	.602	.307	.783	.654	.887	.680	.965	.912
Air Filter	.419	.077	.524	.228	.731	.245	.848	.580
Use of Curtains	1.156	.740	1.752	.338	.653	.075	.729	.255
Tile Floor	1.067	.850	1.899	.141	1.027	.905	1.329	.261
Wooden Floor	1.171	.615	1.571	.220	.937	.757	1.037	.878
Carpeting	.269	.204	.393	.386	1.037	.928	1.371	.461
Pet at Home	.885	.779	.904	.850	.946	.842	.998	.995
Breast Feeding ≥1m	.706	.283	.618	.199	.951	.817	.908	.696

Abbreviations: UVA: Univariate Analysis; MVA: Multivariate Analysis.

### Co-existing of risk factors for the development of AD, AR or AS in children from non-atopic parents

We performed several analyses to investigate co-existing risk factors for the development of AD, AR or AS. We selected the risk factors individually associated with AD, AR or AS, and calculated the odds ratios (OR) for two or three coexisting risk factors for the development of allergic diseases based on the ratio of affected children in the risk group versus those in the non-risk group. Coexistence of breastfeeding and C/S revealed the highest risk for AD (OR=3.11, p=.006), and the coexistence of breastfeeding, family member smoking and tile flooring also revealed a significant predictor for AD (OR=2.77, p=.030). The coexistence of family member smoking, use of curtains, and tile flooring was a significant predictor for AR (OR=2.11, p=.013), and combinations of either two of the three factors all presented statistical significance (Table 5). Living with curtains but without air filters was a significant predictive factor for AR (OR=2.65, p<.001). In AS children from non-atopic parents, we found that male gender without the use of air filter was a significant predictive factor for AS at OR=1.93 (p=.039). Male gender and living in a townhouse concomitantly predicted AS with an OR of 1.91 (p=.045). Coexistence of male gender, family member smoking, and living in a townhouse had an OR at 2.263 but did not reach a level of significance (p=.063) because only a small number in the study population were exposed to all three risk factors (Table 5).

**Table 5:** Coexisting risk factors for the development of AD, AR or AS in children from non-atopic parents

	Coexisting risk factors			N1/N2	OR	*p*
**AD**						
	Family smoking+	Breastfeeding+		59/310	1.957	.125
	Family smoking+		Tile flooring+	61/303	1.507	.367
		Breastfeeding+	Tile flooring+	170/189	2.158	.049
	Family smoking+	Breastfeeding+	Tile flooring+	38/319	2.776	.030
	C/S+	Breastfeeding+		55/315	3.111	.006
	C/S+		Dehumidifier+	15/352	1.714	.492
		Breastfeeding+	Dehumidifier+	42/317	2.442	.055
	C/S+	Breastfeeding+	Dehumidifier+	6/352	2.147	.492
**AR**						
	Family smoking+	Curtaining+		77/287	1.875	.018
	Family smoking+		Tile flooring+	61/303	2.107	.009
		Curtaining+	Tile flooring+	222/146	1.787	.015
	Family smoking+	Curtaining+	Tile flooring+	54/310	2.107	.013
	Male gender	Air filter-		167/201	1.925	.004
	Male gender		Curtaining+	174/194	1.905	.005
		Air filter-	Curtaining+	240/128	2.647	.000
	Male gender	Air filter-	Curtaining+	138/230	2.087	.001
**AS**						
	Male gender	Air filter-		167/201	1.930	.039
	Male gender		Curtaining+	174/194	1.447	.239
		Air filter-	Curtaining+	240/128	1.650	.157
	Male gender	Air filter-	Curtaining+	138/230	1.555	.160
	Male gender	Family smoking+		61/315	1.473	.317
	Male gender		Housing (Townhouse)	134/215	1.914	.045
		Family smoking+	Housing (Townhouse)	65/281	1.762	.127
	Male gender	Family smoking+	Housing (Townhouse)	35/311	2.263	.063

Notes: Coexisting risk factors are selected from those representing individual association with AD, AR or AS, and the odds ratio (OR) for the 2 or 3 combined risk factors on the development of allergic diseases are calculated based on the affected children in the risk (N_1_) versus non-risk groups, (N_2_)_,_ where N_1_ is number of subjects with all listed risk factor and N_2_ is number of subjects not with all listed risk factors. “Air filter-“ is regarding to no use of air filter at home.

### Risk factors for comorbidities of AD, AR and/or AS in children from non-atopic parents

In the group with a comorbidity of “AD and/or AR”, significant risk factors were male gender (OR=1.90, p=.024) and tile floor use (OR=2.14, p=.019). Air filter use showed a significant protective factor (OR=0.449, p=.032) for the comorbidity of “AD and/or AR”. Analysis of the comorbidity of “AR and/or AS” showed that male gender (OR=2.07, p=.011), use of curtains (OR=2.56, p=.030), and tile floor use (OR=2.38, p=.008) were significant risk factors. In the patients with a comorbidity of “AD and/or AR and/or AS”, male gender (OR=1.83, p=.029) and tile floor (OR=2.08, p=.020) raised a significant risk. Use of air filter lowered the risk with a borderline significance (OR=0.50, p=.053) for the comorbid allergic diseases. Overall, male gender, use of curtains and/or tile floor are risk factors for comorbidity of allergic diseases, and use of air filters is a protective factor from comorbidity of allergic diseases (Table 6). Although the risk factors of AR and AS were not the same based on the statistical significant analyses, they revealed higher odds ratios of certain risk factors to AR and AS, such as family smoking (OR=1.528 and 1.199, p=.084 and .597), and use of curtain (OR=2.062 and 1.156, p=.035 and .740). On the other hand, use of air filter revealed lower ORs to AR (OR=.427, p=.008) and AS (OR=.419, p=.077).

**Table 6:** The risk factors for comorbidity of allergic diseases in children from non-atopic parents

	AD and/or AR		AD and/or AS		AR and/or AS		AD and/or AR and/or AS	
	OR	*P*	OR	*P*	OR	*P*	OR	*P*
Gender (Male)	1.900	.024	1.146	.669	2.074	.011	1.829	.029
Preterm	.338	.115	.436	.301	.471	.235	.430	.179
Smoking	1.719	.075	1.001	.997	1.798	.055	1.563	.135
Breast Feeding ≥1m	.933	.816	1.084	.813	.730	.288	.807	.461
C/S	1.248	.483	1.083	.828	1.172	.616	1.215	.529
First Parity	.707	.226	.775	.435	.711	.237	.683	.173
Housing (Townhouse)	.801	.456	1.095	.792	.762	.361	.830	.523
Dehumidifier	.597	.212	.966	.938	.476	.079	.601	.204
Air Filter	.449	.032	.519	.130	.507	.066	.500	.053
Wooden Floor	1.573	.109	1.736	.088	1.420	.216	1.424	.202
Carpeting	.491	.314	.280	.235	.445	.257	.425	.223
Use of curtains	1.976	.104	1.213	.682	2.561	.030	1.993	.088
Tile Floor	2.140	.019	1.535	.243	2.380	.008	2.075	.020
Pet at Home	1.211	.622	1.295	.553	1.107	.794	1.019	.961

Abbreviations: AD, atopic dermatitis; AR, allergic rhinitis; AS, asthma. OR and p values are calculated by multivariate analysis

## DISCUSSION

This study found that the prevalence of AD, AR and AS was, respectively, 8.2, 30.8 and 12.4% in the children of non-atopic parents. The prevalence is not significantly different from that in children of one parent atopic. The results support that certain environmental factors affect development of AD, AR and/or AS in children of non-atopic parents. In children of non-atopic parents, breastfeeding for more than 1 month and C/S were significant risk factors for development of AD, while male gender, exposure to a family member who smokes and tile flooring were significant risk factors for development of AR. The combination of male gender and no use of air filter, or male gender and living in a townhouse, posed a significantly higher prediction for AS.

Although breastfeeding has been reported to be protective against allergic diseases [21–23], we found that breastfeeding for more than one month revealed an insignificantly lower OR of protection from AS (0.618) or AR (0.868) in children from non-atopic parents. This result is similar to a recent cohort study from Copenhagen Prospective Study on Asthma which showed that breastfeeding does not protect against allergic sensitization in early childhood and allergy-associated disease at age 7 years [24]. Another cohort study from Japan also found that breastfeeding, whether exclusive or not, was not significantly correlated to protection from wheezing and asthma [25]. Moreover, we found that breastfeeding was a significant risk factor for AD in children from non-atopic parents. In the Copenhagen cohort study, they also showed an increased risk of eczema but reduced risk of early wheezing disorder in exclusively breastfed high-risk infants. The New Zealand cohort study found that a longer duration of breastfeeding significantly elevated the risks of developing AD in children at 3.5 years of age and concluded that breastfeeding should not be recommended to protect against the development of AD [26]. An Osaka Maternal and Child Health Study investigated the relationships between breastfeeding and atopic eczema in Japan, and found that exclusive breastfeeding for more than 4 months (OR 2.41, 95% CI 1.10-5.55) or partial breastfeeding for more than 6 months (OR 3.39, 95% CI 1.20-12.36) significantly increased the risk for the development of atopic eczema in children of parents without allergic history [27]. Yu and coworkers [28] studied the relationships between C/S and AD and found a significant higher risk of AD for those children born by C/S (OR=1.50, 95% CI =1.01-2.22). These results corresponded to our study, indicating breastfeeding and C/S increased the risk for the development of AD in children from non-atopic parents, while these two factors were not significantly associated with development of AD in children with atopic parents. This suggests that children from parents without allergic history are more susceptible to influence of environmental factors such as breastfeeding that might affect microbiota of gastrointestinal (GI) tract, and C/S that could also affect microbial colonization in GI tract and on the skin, highlighting the impact of early microbial colonization on the perinatal prevention of AD in children from non-atopic parents.

The present study also found that preterm delivery appeared to be a protective factor of allergic diseases because the odds ratios were 0.602, 0.462 and 0.750, respectively for AD, AR and AS. A significant protection (p=.046), however, is only found in children with AR but not AD or AS. This may be attributed to the fact that the prevalence of AR is higher than that of AD or AS. Crump et al. [29] also conducted a large nationwide cohort study to study the relationships between gestational age and risk of AR. They found that a lower gestational age is associated with a lower risk of AR. Taken together from our and others studies, we postulate a possible prematurity protection of AR related to early exposure to maternal flora or pathogens for the immune regulation on the development of allergic diseases.

In addition, male gender was a significant risk factor for AR in children from non-atopic parents. The result was consistent with many studies reporting a higher prevalence of allergic diseases and higher total IgE levels in boys than females [30]. In another review article, Johnson et al. reported that boys had more wheezing and fewer respiratory tract infections during infancy than girls in Christchurch Health and Development Study in New Zealand. Similarly, boys’ total IgE levels increased faster than girls’ between birth and 2 years of age in Detroit Childhood Allergy Study [31]. In another study, however, gender was found to be an insignificant predictor for ever-diagnosed AS after controlling confounding factors [32]. In this study, we found that male gender was a risk factor to development of AR, and the combination of male gender and use of curtains with or without use of air filter increased the odds ratios. In contrast, we did not find that male gender alone was a significant risk factor to AS unless there was coexistence of living without air filter.

Our study also found that exposure to a family member who smokes was a significant risk factor (OR=1.89) for AR. The combination of exposure to a family member who smokes and tile flooring at home increased the risk for AR in children from non-atopic parents while it is not a significant factor for AS both in univariate and multivariate analyses. This suggests that the impact of perinatal tobacco smoke exposure on the development of allergic diseases did not appear consistent in AS. Ponsonby, et al. [33] showed that exposure to environmental tobacco smoke in infancy was related to subsequent occurrence of wheeze or AS. Another study reported that maternal smoking during pregnancy was one of the predictors of two or more wheeze episodes in the first year of life. However, the Childhood Allergy Study in Detroit found that neither maternal nor paternal smoking was associated with IgE level in cord blood [31], suggesting perinatal tobacco exposure influences remodeling of airway allergic diseases but not IgE production. As we defined AR or AS based on both physician-diagnosed history and presence of allergen-specific IgE, the risk factors identified for AR or AS may be related to IgE production and airway remodeling. The facts that coexisting of family member smoking and tile flooring increased the risk to AR and coexisting of family member smoking and living without air filter increased the risk to AS in our cohort study at subtropical country suggest that prediction and prevention of airway allergic diseases should be different based on the environment, weather and culture. Further studies are needed to validate the impacts of flooring, air filters and tobacco exposure on AD, AR and AS in different global regions.

### Strengths and limitations of the present study

This large scale study recruited more than one thousand newborns prenatally, and conducted long-term followed-up for more than 6 years. We adopted stringent definitions for parental and childhood allergic diseases, based on clinical features of physician's diagnosis combined with the detection of total and specific IgE levels. Given the detailed information collected, this birth cohort enabled us to include parental and perinatal environmental risk factors for the analyses on the development of allergic diseases from birth to 6 years of age. There are some limitations of the study. Questionnaires were used to collect the participants’ information, which may raise recall bias due to interpersonal reporting bias of parents resulting in over- or under-estimation of the actual risks. Some of the items in the questionnaire were reported dichotomously. Dichotomous responses were unable to be interpreted in dose-response relationships between risk factors and allergic diseases. For example, number of pets kept, hours of daily dehumidifier use and air filter use are not clarified for the analyses. Similarly, severities and dynamics of allergic diseases are also not recorded. The children were followed up to 6 years of age, and some allergic diseases may not have been fully developed in some subjects. The birth cohort was conducted in a medical center in southern Taiwan where is located in a subtropical region. This could limit the generalization of the results to other parts of the country or the world.

In conclusion, prevalence of AR (p<.001) and AS (p=.018) was significantly higher in children from atopic parents than those from one atopic or non-atopic parents, suggesting children of parents who are both atopic develop atopic diseases more related to inherited causes. The prevalence of allergic disorders in children of non-atopic parents is similar to those with one atopic parent, indicating children of non-atopic parents develop atopic diseases as a result of environmental influences. Atopic dermatitis is a disease somehow different from airway allergic diseases in term of different inheritance (Table 2) and environmental risk factors (Table 4). Avoidance of breastfeeding and C/S may rescue AD, and avoidance of curtains and use of air filters may decrease AR and AS in children from non-atopic parents in a subtropical region. The interactions among atopic parents, non-atopic parents and environmental factors in different global regions on the development of allergic diseases require further studies to validate the risk or protective factors for early perinatal prevention of allergic diseases.

## MATERIALS AND METHODS

### Design of the study

The study is derived from a birth cohort longitudinally followed for up to 6 years of age. The birth cohort was described in detail in previous works [4, 7, 17, 34, 35]. In brief, 1,848 newborns were recruited prenatally between December 1998 and April 2009, crossing 11 years. The potential confounders due to changes of environmental situations during the 11 years are possible. However, we analyzed the demographic data between the early enrolled population (1998-2003) and the late enrolled population (2004-2009) showed no significant differences (mode of delivery (p=.645), gestational age (p=.933), and gender of the infants (p=.091), suggesting the situation bias may be insignificant. One thousand four hundred and ninety seven infants had cord blood samples, 1,236 and 756 children completed their 3-year and 6-year follow-up, respectively, and 737 children completed all charts and blood collection for final analysis. Informed consent was obtained from the expectant parents during their prenatal visits to an obstetric clinic at a large teaching hospital in southern Taiwan. The ethical approval was given by the Institutional Review Board of the hospital. Atopic history and blood samples from the mothers and fathers were collected during the third trimester of pregnancy. Clinical examination, questionnaire, and blood samples for total and specific IgE of the children were collected at each follow-up visit.

### Sample collection and measurements

Umbilical cord blood samples were collected immediately after the infants were born. Birth history, including gestational age, mode of delivery (normal delivery or Caesarean-section), and gender, was collected by chart review. Total and/or specific IgE levels were measured in umbilical cord blood and during follow up visits. Levels of specific and total IgE levels were determined by Pharmacia CAP system (Pharmacia & Upjohn Diagnostic AB, Uppsala Sweden) as described previously [4, 34]. Cord blood total IgE levels were assessed using a low-range total IgE detecting system while parental total IgE levels and follow-up total IgE levels of the children were determined using a full-range total IgE detecting system. Elevated cord blood IgE levels were defined as any level greater than 0.5 kU/L. Sensitization to aeroallergens was defined as a specific IgE level of greater than 0.35 kU/L, and sensitization to food allergens was defined as a specific IgE level of greater than 0.7 kU/L.

### Follow up progress and definitions of parental atopy & childhood allergic diseases

Data of environmental factors were collected during follow-up in obstetric and pediatric clinics utilizing questionnaires. On recruitment, a questionnaire about parental allergic history, including AD, AR, and AS, and parental smoking were collected in the second or third trimester of pregnancy. At delivery, the information about the mode of delivery, gestational age, parity, and gender of the infant was recorded. Infants at 6 and 18 months of age were followed up to collect their breastfeeding and infection history, housing conditions with and without townhouse, dehumidifier, air filter, curtaining, carpeting, pets, and tile vs. wooden floor, and information on atopic eczema, cough or sneezing without colds, and audible wheezing episodes. Children at 3 and 6 years of age, questionnaires were distributed and performed by a pediatric allergist to collect the information about physician-diagnosed allergy diseases, including AD (chronic or relapsing dermatitis with erythematous, scaly or itchy rashes on the face, neck, anterior chest wall, extensor areas or the flexural folds of extremities), AR (sneezing or running nose in the morning or night over 2 weeks in the absence of infection, and diagnosed by a physician), and AS (recurrent at least 3 episodes of wheezing, exercise-induced cough or coughing in the morning or night over 2 weeks, and diagnosed by a physician).

We defined parental atopy by a history of AD, AR, or AS together with either a positive CAP or a total IgE level ≥ 100 kU/L. The CAP test was considered positive for food allergen ≥ 2 tiers (0.7 kU/L) or aeroallergen ≥ 1 (0.35 kU/L) tier. The children completing the 6-year-old follow-up were categorized into 3 groups: neither parent with atopy (n=380), one parent with atopy (n=293), or both parents with atopy (n=64). A childhood allergic disease was defined by a clinical diagnosis of allergic disease of AD, AR, or AS together with aeroallergen sensitization at specific IgE levels ≥ 0.30 kU/L or food allergen sensitization at specific IgE levels ≥ 0.75 kU/L. Clinical diagnosis of AD is defined by physician-diagnosed AD and characteristic distribution of dermatitis for more than 2 weeks in recent 6 months; clinical diagnosis of AR is defined by physician-diagnosed AR and characteristic features with morning and/or night sneezing for more than 2 weeks in recent 6 months; clinical diagnosis of AS is defined by physician-diagnosed AS and characteristic features with exercise-induced cough or morning and/or night cough for more than 2 weeks in recent 6 months.

### Statistical analysis

We compared the distribution of demographic data between children with and without completion of the follow-up by Chi-square test. We analyzed the prevalence of allergic disorders among children of parents who are either both atopic, one is atopic or both are non-atopic by Chi-square test. Then we examined the relationships between the risk factors and the development of AD, AR, and/or AS of children from non-atopic parents by univariate logistic regression analysis (UVA) and multivariate logistic regression analysis (MVA). To better understand coexisting risk factors for the development of AD, AR or AS in children from non-atopic parents, we selected the risk factors individually associated with AD, AR or AS, and calculated the odds ratios (OR) for the 2 or 3 coexisting risk factors on the development of allergic diseases based on the ratio of affected children in the risk group (N1) versus the ratio of those in the non-risk group (N2), where N1 is the number of subjects with listed risk factors and N2 is the number of subjects without listed risk factors. A p-value of less than .05 was considered statistically significant. All statistical analyses were carried out by SPSS statistical software package.

## Abbreviations

AD: atopic dermatitis
AR: allergic rhinitis
AS: asthma
IgE: immunoglobulin E
C/S: Cesarean section
UVA: univariate analysis
MVA: multivariate analysis
